# Modular mechatronic system for stationary bicycles interfaced with virtual environment for rehabilitation

**DOI:** 10.1186/1743-0003-11-93

**Published:** 2014-06-05

**Authors:** Richard G Ranky, Mark L Sivak, Jeffrey A Lewis, Venkata K Gade, Judith E Deutsch, Constantinos Mavroidis

**Affiliations:** 1 Biomedical Mechatronics Laboratory, Department of Mechanical & Industrial Engineering, Northeastern University, 360 Huntington Avenue, Boston, MA 02115, USA; 2 VRehab LLC, Jersey City, NJ, USA; 3 RiVERS (Research in Virtual Environments & Rehabilitation Sciences) Laboratory, Department of Rehabilitation and Movement Sciences, Rutgers Biomedical and Health Sciences, 65 Bergen Street, Newark, NJ 07101-1709, USA

## Abstract

**Background:**

Cycling has been used in the rehabilitation of individuals with both chronic and post-surgical conditions. Among the challenges with implementing bicycling for rehabilitation is the recruitment of both extremities, in particular when one is weaker or less coordinated. Feedback embedded in virtual reality (VR) augmented cycling may serve to address the requirement for efficacious cycling; specifically recruitment of both extremities and exercising at a high intensity.

**Methods:**

In this paper a mechatronic rehabilitation bicycling system with an interactive virtual environment, called Virtual Reality Augmented Cycling Kit (VRACK), is presented. Novel hardware components embedded with sensors were implemented on a stationary exercise bicycle to monitor physiological and biomechanical parameters of participants while immersing them in an augmented reality simulation providing the user with visual, auditory and haptic feedback. This modular and adaptable system attaches to commercially-available stationary bicycle systems and interfaces with a personal computer for simulation and data acquisition processes. The complete bicycle system includes: a) handle bars based on hydraulic pressure sensors; b) pedals that monitor pedal kinematics with an inertial measurement unit (IMU) and forces on the pedals while providing vibratory feedback; c) off the shelf electronics to monitor heart rate and d) customized software for rehabilitation. Bench testing for the handle and pedal systems is presented for calibration of the sensors detecting force and angle.

**Results:**

The modular mechatronic kit for exercise bicycles was tested in bench testing and human tests. Bench tests performed on the sensorized handle bars and the instrumented pedals validated the measurement accuracy of these components. Rider tests with the VRACK system focused on the pedal system and successfully monitored kinetic and kinematic parameters of the rider’s lower extremities.

**Conclusions:**

The VRACK system, a virtual reality mechatronic bicycle rehabilitation modular system was designed to convert most bicycles in virtual reality (VR) cycles. Preliminary testing of the augmented reality bicycle system was successful in demonstrating that a modular mechatronic kit can monitor and record kinetic and kinematic parameters of several riders.

## Background

Cycling has been used in the rehabilitation of individuals with both chronic conditions such as stroke [1,2], multiple sclerosis (MS) [3] and chronic obstructive pulmonary disease [4] as well as post-surgical populations such as heart [5], hip [6] and knee surgery. The proposed and partially documented benefits of cycling are many and include improved aerobic fitness [5,7], increased muscle strength [1,8,9] and even transfer to other activities such as walking [7,8]. Cycling has been performed in isolation, or in combination with electrical stimulation [1,7,8], and augmented with virtual reality [10].

A classic presentation for individuals with both chronic and post-surgical conditions is lower limb asymmetries in strength, coordination and functional use. These asymmetries have been documented for individuals with MS [11] and unilateral total hip replacement [12]. Asymmetries have also been identified in stair climbing for individuals with osteoarthritis (OA) of the knee that are asymptomatic [13]. Furthermore, when individuals with motor control asymmetries bicycle for rehabilitation they do so with an asymmetrical pattern. This has been shown for various populations such as individuals with anterior cruciate ligament deficiency [14] as well as individual post-stroke [15]. These difficulties are in part reversed when cycling is coupled with functional electrical stimulation (FES). However, provision of FES is not always possible. Therefore, among the challenges with implementing bicycling for rehabilitation is the recruitment of both extremities, in particular when one is weaker or less coordinated.

Feedback in the form of virtual reality augmented cycling may serve to address the requirement for efficacious cycling; specifically recruitment of both extremities. Bicycling systems interfaced with virtual reality augmentation are few. They have been used to improve sitting balance and symmetry [16] and assessed for their psychological benefits to the riders [10]. A bicycling system augmented by virtual reality has not been used however to promote *limb* symmetry.

Innovations in bicycle hardware have facilitated a more realistic cycling experience by increasing the range of motion of the stationary bicycle or handles [17-22]. Mechanical linkages and dampers allow the handles and bike frame to lean in the coronal and transverse planes to simulate uneven rocky terrain. Developments to address interfacing existing exercise equipment with a computer or electronic device to either translate the rider’s actions as an all-purpose controller or specifically copy their motions into a virtual environment using selected gains have been reported in [23-25]. Heart rate as a surrogate for the rider’s level of exertion has been used in isolation to control the difficulty of a game interfaced with the bicycle [26]. The interfacing of a Virtual Environment (VE) with bicycle however, has not been approached from a multi-modal perspective where physiological and biomechanical measurements are combined and applied to impaired participants.

Instrumented bicycle pedals have been used in evaluating kinetic/kinematic capabilities for subjects with both healthy and plegic lower extremities [27-31]. Experimental setups for pedal force sensing have involved a variety of strain-gauge based designs [32] and piezoelectric elements [30,33]. Using an inertial measurement unit (IMU) for detecting pedal angle has not been used before in a clinical setting for stroke rehabilitation. In stationary bicycle pedals the most frequent angle detection methods have been mechanical [34] or optical-encoder based [20,27]. An IMU requires no hardware linkage connections which means decreased mechanical complexity and likelihood of component failing.

Adding games to stationary bikes has been used to create several virtual reality cycling systems [17,18]. These systems were designed for fitness of active individuals, rather than rehabilitation of fitness and motor control deficits of individuals with disabilities. Representative existing systems are prohibitively expensive for a rehabilitation population and provide insufficient feedback to the user. Those systems with proprietary software have the potential to transmit exercise information to the screen and to store information, while others can only drive existing games, controlling only speed or direction. While these systems can perform well for healthy individuals, most of them are too expensive for small clinics and homes.

Although there has been extensive design evolution on bike pedal instrumentation, there has been limited research on incorporating handle bar sensors alongside the pedal sensors for assessing the gripping forces. Furthermore, there are no sensorized exercise bicycle systems that are modular and have the capability of using physiological (heart rate) and biomechanical (kinetics and kinematics) inputs to drive a virtual environment while at the same time collecting performance data. Evaluation of the current commercially comparable devices necessitates a low cost, state of the art system with diverse measurement functionality, immersion, and adaptability to any current stationary bicycle.

In this paper the Virtual Reality Augmented Cycling Kit (VRACK), a virtual reality mechatronic bicycle rehabilitation system is presented. VRACK was designed as a modular system that can convert most bicycles into virtual reality (VR) cycles. Novel hardware components embedded with sensors were implemented on a stationary exercise bicycle to integrate physiological and biomechanical parameters of participants immersed in a virtual environment (VE) providing the user with visual, auditory and haptic feedback. This modular and adaptable system attaches to commercially-available stationary bicycle systems and interfaces with a personal computer for simulation and data acquisition processes. Among the attributes of the VRACK is bike navigation task in which force transducers in the pedals are linked to the verticality of the rider, specifically designed to promote symmetry. In addition heart rate monitoring and feedback are used to promote exercise intensity suitable for health and fitness.

### System overview

**Figure 1:**
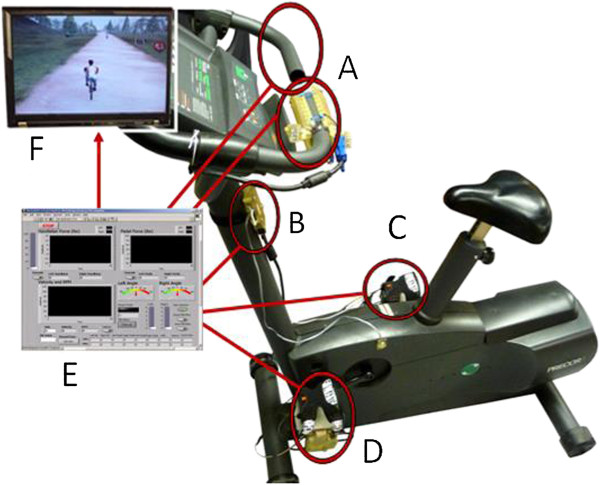
**Bicycle system complete overview: A: handlebar module; B: heart rate monitor; C, D: pedal modules; E: practitioner interface; F: virtual environment.***Copyright Rivers and Mechatronics Labs*.

The virtual reality augmented cycling kit (VRACK), shown in Figure 1, consists of novel hardware components embedded with sensors that are used to enhance the use of a typical stationary exercise bicycle. Its modular sensor system has been mounted to a standard exercise bicycle as seen in Figure 1. The complete instrumentation includes two sensorized handlebar modules, two instrumented pedal modules, a heart rate monitoring system, and additional electronics for signal conditioning, power, and connection to a laptop via USB and an interactive virtual environment designed for rehabilitation. The parameters monitored by these systems are communicated to a practitioner’s interface to customize the therapeutic intervention and monitor quantitative performance of each parameter.

In the virtual environment, a pace rider is displayed as a visual target to motivate the patient. The patient is instructed to catch the pace rider, who rides at the patient’s target heart rate (HR). Previous systems have not used this dynamic velocity tool for HR and used either fixed HR to induce a level of patient exertion or HR scaling to level the playing field between human players of different fitness level [35]. The RE07L Wireless Receiver Module and T31 coded elastic chestband (Polar Electro Inc., Lake Success, NY, USA) was selected for HR measurement. The chest-band is worn during exercise with the transmitter in skin contact just below the center of the sternum, and outputs a pulse for each heart beat.

### Instrumented handlebar module

**Figure 2:**
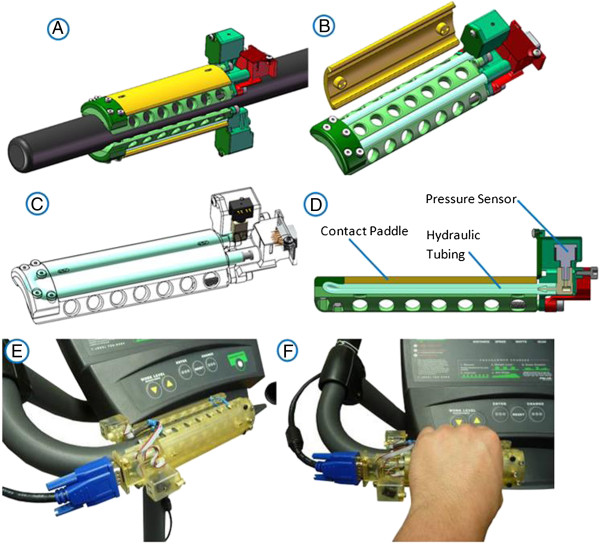
Instrumented handlebar: A: top & bottom handle surface on bike frame; B: CAD model compression with paddle lifted; C: View of functional components; D: physical prototype of handlebar top; E: handle prototype mounted on stationary bicycle; F: Handle in Operation.

Riders may have motor control and sensory deficits, which impact their ability to comfortably grasp the bicycle handlebar. Combined with the range of hand anthropometries this can be challenging for designs that use discrete points or even dense arrays of force sensing elements. The handle system in Figure 2 is a novel type of hydraulic dynamometer, which measures applied force to control dynamic motion of the rider in the virtual environment. This module has two sensing surfaces on each hand to provide information to the practitioner *virtual interface* (VI) of which hand and which side of that hand is applying a greater force. This avoids the convolution in determining whether the affected side is exercising or is being overpowered by the unaffected side, as is the case with some previous work which combines all of these variables into a single net torque measurement about the front fork. The handle housing was designed with a diameter to provide the greatest ergonomic comfort for grasping whilst allowing the user to comfortably maximize their isokinetic strength [36-38].

The surfaces contacting the tubing are designed after a simply supported beam where the sum of the support reaction forces equals the loads from the hand as the tubing compression forces. This tubing configuration is designed to keep the paddle contacting the hand self-balancing since it is supported by two sections of the same chamber. This way a force applied at any location between the tube sections is distributed evenly across the entire chamber and transmitted to the hydraulic pressure sensor (Model PX 35, Omega Engineering). The liquid inside the tube is a medium-density mineral oil which is non-reactive and stable for ranges of room temperature.

### Instrumented pedal module

**Figure 3:**
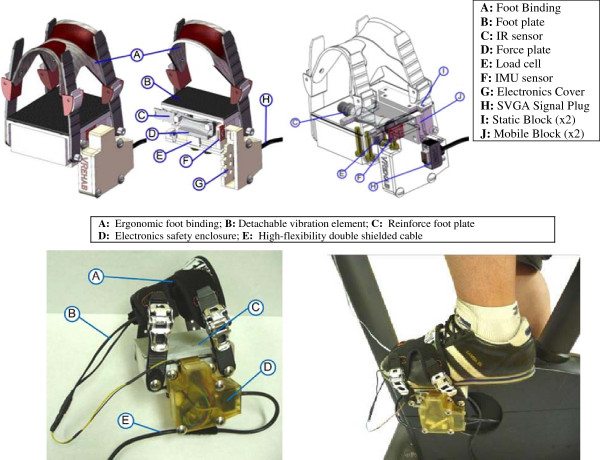
Instrumented pedal module CAD and cross-sectional view (top); Prototype and prototype in operation (bottom).

Asymmetry in pedalling is a frequent presentation following conditions such as a stroke that predominantly affect one side of the body. It is important to independently measure each foot to eliminate the unaffected side overcompensating for the affected side.The pedal module, shown in Figure 3, is designed to attach to the crankshaft of a bicycle, stationary bicycle. This system does not require any specialized footwear from the rider, is adjustable to a range of shoe sizes, and covers more surface area than conventional cleated pedals to constrain the foot. Flow Flite 4 snowboard bindings (Flow, San Clemente, USA) provide attachment to the pedal. These adjust across the dorsal side of the wearer’s foot from the base of the internal and middle cuneiform down to the middle of the metatarsals. Two different sizes of bindings and ratcheting buckles accommodate two groups of anthropometric variability.

The forces on the plate are measured by a single-axis low profile compression load cell (LC302-500, Omega, Stamford, CT, USA). Four bolt & spring assemblies provide a collective 50 lb (222.4 N) pre-load compression on the pedal system to enable the single-axis load cell to detect tensile forces in the pedal. The resulting offset for the load cell voltage is zeroed in software. This enables measurement of tensile force up during pedalling to this pre-load max, and compressive forces up to 450 lbs (2001.7 N).

The pedal tilt of the ankle is monitored by an inertial measurement unit (IMU), which contains an accelerometer and gyroscope to detect tilt in dorsi and plantarflexion. The raw data from the accelerometers and gyroscope are collected from the practitioner interface and then analysed using a Kalman Filter [39,40]. The rate gyroscopes are used to determine the angular velocity (ω) of the crank corrected each revolution by the infrared interrupters. Infrared reflectors (IR) were implemented to control the drift of the IMU as well as measure the number of rounds per minute (RPM) of the crank. Every time the crank arm passes in front of the pedal-mounted sensor, this indicates that the pedal is perpendicular to the crank which is used as a reference to zero the drift from the IMU. At the same time, two small IR sensors are mounted on the body of the stationary bike, one to face each pedal. These body-mounted IR posts detect the number of times the crank passes the top-dead-center position and thus calculate the RPM.

To provide haptic feedback to the rider’s feet, vibration elements (Precision Microdrives 310–101, Precision Microdrives, London, UK) were implemented in the pedal bindings. Two of these elements were encased and attached to the inside of the bindings with Velcro so can be relocated anywhere on the dorsal shoe surface. They are activated manually to augment sensory input to the foot.

### Data acquisition & user interfaces

**Figure 4:**
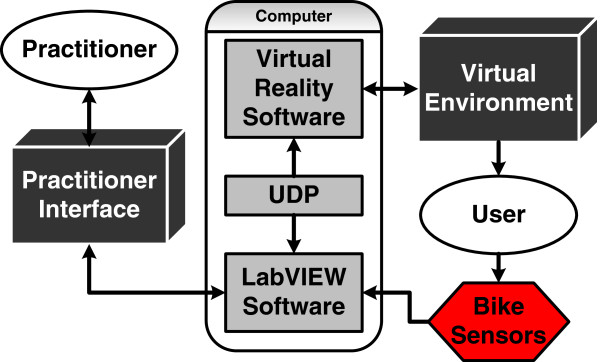
Information communication diagram.

A LabVIEW Virtual Instrument (VI) was created for the sensors of the bike system to interface with the Virtual Environment (VE). Signals are acquired using a PCI DAQ card for hardware timing, this allows for more reliable timing between the discrete signals being gathered. The sampling frequency is 500 Hz, with a cut-off frequency of 250 Hz. Low-pass analogue RC filters and digital Chebyshev filters were used to condition the signals from the pedal load cells, pedal IMUs, and handlebar pressure sensors. The communication between the VI and the Virtual Reality Software was achieved utilizing the User Datagram Protocol (UDP). The flow of information throughout the system is outlined in Figure 4.

#### ***Practitioner interface***

**Figure 5:**
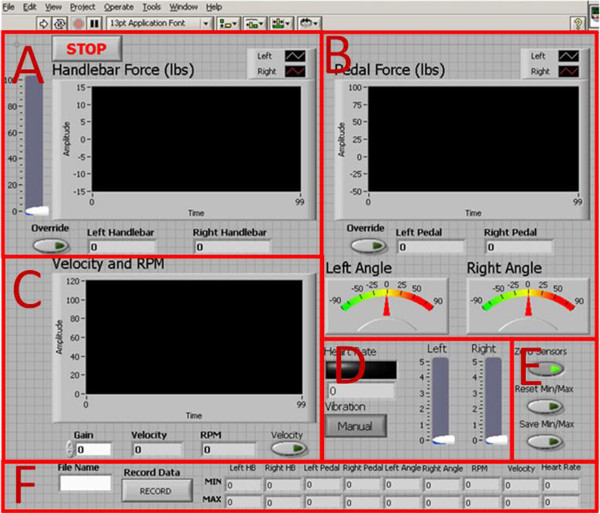
**The Practitioner Interface (PI) allows quantitative viewing of the sensors’ data and complete control to scale & activation for when they are sent to the Virtual Environment (VE).** Signals are grouped for handlebars **(A)**, pedals **(B)**, crank RPM **(C)**, heart rate & vibration **(D)**, sensor tare to zero resting measurement **(E)**, and data logging **(F)**.

The Practitioner Interface (PI) displays simultaneously all measured signals from the sensor modules and allows complete control over their logging and activation/translation to the virtual environment for the patient. This ability to retain selected inputs and ‘autopilot’ the others allows the patient’s exercise regime to become customized to focus on key neural and musculoskeletal parameters. Upper extremity motor function can be trained with just the handlebars steering through the course, with a fixed pedal velocity. Focus on lower extremity symmetry retraining, is achieved by deactivating the handlebars and concentrating only on pedal force symmetry. For riders with impaired upper extremity isokinetic grip strength the gain of the handlebar signal can be increased. The PI is shown in Figure 5.

Each handlebar has a single signal displayed on the PI for the magnitude of the net force, but the near and far surfaces of each handle are being recorded separately and summed for the net force. The sign of this magnitude is the direction indicating towards or away from the rider. The filler bar is used to adjust the gain of the handlebars impact in the VR simulation. The scaling operation takes place after being displayed in the PI and only affects the VE.

Each pedal has its output displayed as a single net force, with the sign indicating compression or tension. Patients with lower extremity strength or coordination asymmetries cannot isolate the performance of the unaffected side compensating for the affected side. Since current stationary bikes (and also higher-end systems) record output power measurements from the flywheel at the middle of the two bonded crank arms this convolutes the output. Separating pedal force sensing components for each foot is the only way to remove this convolution effect of the unaffected side compensating for the affected side. The pedal angle is displayed using two gauges that span from 90° to -90°.

Each heart beat illuminates a light on the PI and heart rate (beats per minute) is displayed. The vibration mode can be toggled between automatic and manual (intensity – frequency controlled by the practitioner individually for each foot). Locating the vibration elements on the surface of the rider’s feet allows testing for sensation of the affected vs. unaffected sides.

#### ***Virtual environment & sensor mapping***

**Figure 6:**
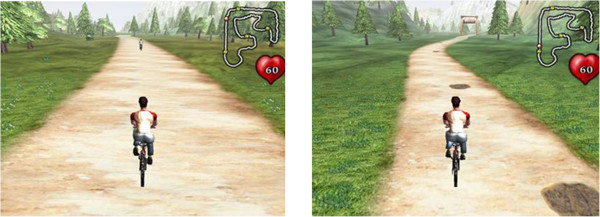
**The virtual rider (in red) must stay with the pace rider (seen ahead on the left figure). ***Copyrighted by the Rivers Lab*.

**Figure 7:**
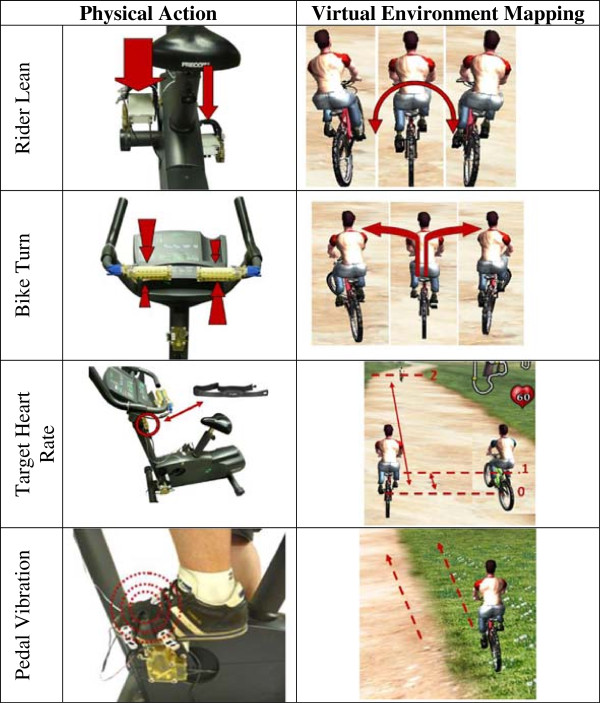
**Functions mapped between the avatar in the VE and the physical rider.***Copyrighted by the Rivers and Mechatronics Labs*.

The purpose of the virtual environment (VE) is to engage the user by providing multi-sensory and performance feedback *as well as shape their motor behavior*. The upper right corner of the simulation displays a map of the virtual environment, and below it the instantaneous heart rate of the rider is displayed (Figure 6). The virtual environment is divided into two regions: the sandy tan path that the user traverses and the green rough that surrounds it. Data are sent from the UDP Sub VI of the interface to the VR simulation to control the virtual rider. The heart rate data control the speed, the handlebar force data control the heading, and the pedal kinetics control the tilt of the rider. *Symmetry of the motor behavior is promoted by displaying a real asymmetry with visual feedback from the rider (tilting to the weaker side when lower extremity forces are asymmetrical on the pedals; or errors in steering with asymmetry of the handle bars). These can be over-ridden completely by the clinician or tempered by manipulating the input gain into the VE*. The dark muddy patches on the trail slow down the rider and may be avoided or trigger the rider to pedal harder. *Vibration is activated in the pedals when the rider is off the path and on the green. This serves as an error signal to correct the cycling path and is consistent with theories about the role of sensory integration being enhanced by processing multi-sensory input.*The practitioner also has the flexibility to customize the VE bike map for course trajectory, path width for rider error margin, and frequency of obstacles for avoidance. Depending on the exercise regime the balance of these elements can focus the patient on a particular motion or exercise. These initial parameters are set on the VR Simulation Menu before a session begins. Figure 7 shows the range of challenge for the course in terms of width, obstacle avoidance, and road curvature. Path width can be adjusted in real time.

Depending on the gear ratio of a real bicycle, the angular velocity of the crank leads to a different linear velocity of the bicycle. Without a gear transmission in the software, a set ratio multiplies the crank velocity. The arc length per time is the multiplication factor for determining linear velocity using Equation 1, where R is the crank radius; θ is the angle change between time measured; t is the time increment:

(1) $$\text{Linear}\;\text{Velocity}=\left(R*\theta \right)/t$$

Within the simulation the handlebars control the trajectory of the virtual rider and differential forces from the left and right handles are subtracted after being acquired in the practitioner’s interface. The final net force steers the virtual bicycle through the simulation. The net force from the two pairs of surfaces will be either a clock-wise or counter clock-wise moment with respect to the position of the front fork of the bicycle. Each of the four sensing surfaces is applied a sign based on the moment it generates. The four individual values are recorded as well as the net from each handle.

For steering within the virtual environment it is important to mimic the reactions from a real bicycle closely to promote user immersion. However, even for a consistent smooth turn there are some oscillations in the handle trajectory, which could be visually disturbing if not set correctly in the simulation visuals. This necessitates an artificial dead zone in the software for the handles to avoid sudden and erratic motions of the virtual rider. Even straight, level pedalling regular motion causes slight oscillations in the upper trunk and handlebar trajectory. The pedalling movement transferred to the arms from the legs has been shown to induce roughly a 2.5° periodic sway even in healthy riders for properly fitted handlebars on a track bicycle [29]. This must be accounted for to determine the dead zone implemented in the software so that it does not affect the data collection, only the visual feedback of the simulation. However this does not have to include the counter-steering effect (occurring during controlled turning of a real bicycle or motorcycle) because the torque experienced prior to the turn is already negligible [41].

The calculations for net handle turning direction and magnitude are shown in Equations 2, 3, 4 while the condition for applying handlebar value to virtual rider is defined by Equation 5:

(2) $$\begin{array}{ll} \left[\text{Left}\;\text{Hand}\;\text{Net}+\text{Right}\right) & \left(\text{Hand}\;\text{Net}]*[\text{gain}\;\text{selected}\;\mathrm{by}\;\text{practitioner}\right]\\ & \;=\text{Turn}\;\text{direction}\;\text{and}\;\text{Magnitude} \end{array}$$

(3) LeftHandNet=LeftFront-LeftBack

(4) RightHandNet=-RightFront+RightBack

(5) |TurndirectionandMagnitude|>DeadZoneValue

### System integration

**Figure 8:**
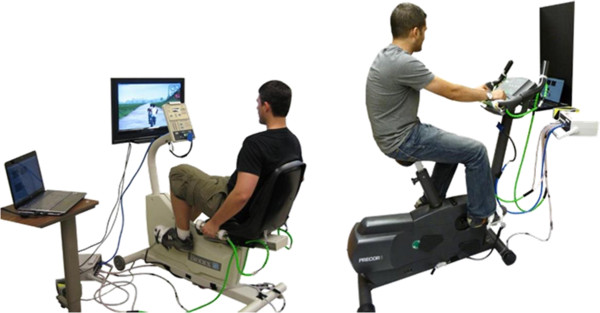
**VRACK system implemented successfully on recumbent (left) and upright (right) exercise bicycles.***Copyrighted by the Rivers and Mechatronics Labs*.

The capability of integrating the VRACK system and its modules in different commercially available exercise bicycles was demonstrated by attaching the handlebar, pedal and heart rate monitor modules in two different bicycles: a recumbent and an upright exercise bicycle (SRC 945–120, Biodex, Shirley, NY; and Precor 842, Precor, Woodinville, WA) as shown in Figure 8. The attachment procedure for all types of exercise bicycles is identical. The instrumented handlebars clamp around the current range of stationary bicycle handlebar diameters using adjustable Velcro fasteners. The raceway of the instrumented pedals uses a 9/16” thread, standard for most stationary exercise bicycle pedals and readily replaces them without specialized tooling. For the instrumented pedal, non-specialized sports footwear may be worn for both types of exercise bicycle. The pedal fit was successfully examined with shoe sizes from 6 to 13 (USA men’s sizes). The performance of the sensorized handlebar module is the same on both types of exercise bike, only the mounting orientation changes as a result of the rider switching to an abducted power grasp when using a recumbent bike. All electrical connections are modular fittings which use standard VGA cables.

## Results

The modular mechatronic kit for exercise bicycles was tested in bench testing and rider tests. Results from these tests are described in this section.

### Bench tests

#### ***Sensorized handlebar dynamic testing***

**Figure 9:**
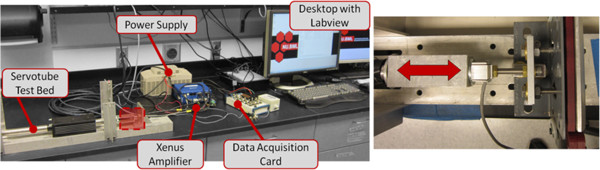
Linear dynamometer system layout with detail at tension load cell interface for dynamic testing of handlebars.

**Figure 10:**
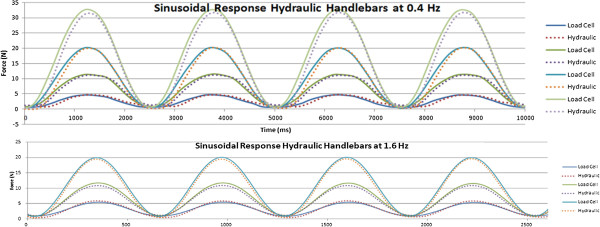
Sinusoidal response of handlebars for 0.4 and 1.6 Hz force profiles.

**Figure 11:**
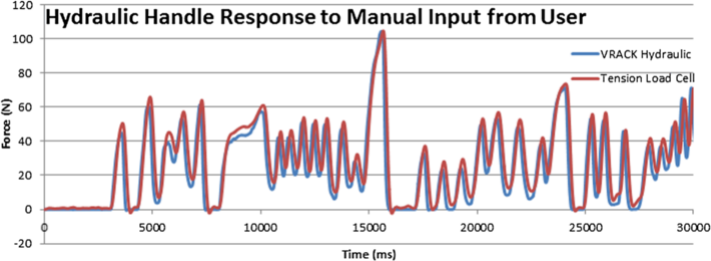
Handlebar sensor data (in blue) can accurately depict forces applied by a human subject as they match the data obtained from the testbed’s load cell (in red).

The sensorized handlebars were calibrated first and then evaluated for dynamic testing using a simple one degree of freedom device where a linear actuator was applying a known, controllable force on the handlebar as shown in Figure 9. The linear dynamometer testbed can apply a static or dynamic force profile using an electromagnetic actuated Servotube (XSL-230-18, Copley Controls, MA) to deliver a compression force to the sensorized unit under testing, with an off-the shelf tensile load cell (Omega Engineering, Stamford, CT) in series for measuring applied input force. The linear actuator was under position or force control depending on the experiments performed. The calibration was carried out by correlating the input force as recorded by the load cell against the output of the handlebar sensors.To validate the calibration procedure of the handlebars we performed a series of dynamic tests that included various periodic force patterns such as sinusoid, square, and sawtooth with varying amplitudes (between 2 and 30 N) and frequencies (between 0.4 to 4 Hz). Each dynamic test lasted 1 minute and the handle was allowed 1 minute between trials to rest. Sample data series obtained from these tests are shown in Figure 10. The data collected using the handlebar sensor matched very well the force curves obtained using the load cell of the test-bed as shown in Figure 10.In addition, we performed a manual test to verify the ability of the handlebar to measure the forces applied by a human subject. With the handlebar still on the testbed, a load profile was applied by hand from a healthy adult male with no previous upper extremity physical deficits. The manual input was a load applied to the servotube by pulling the tension load cell in series with the hydraulic handle. As shown in Figure 11, the forces measured by the handlebar sensor match very closely those measured by the load cell of the testbed.

#### ***Pedal static force tests***

**Figure 12:**
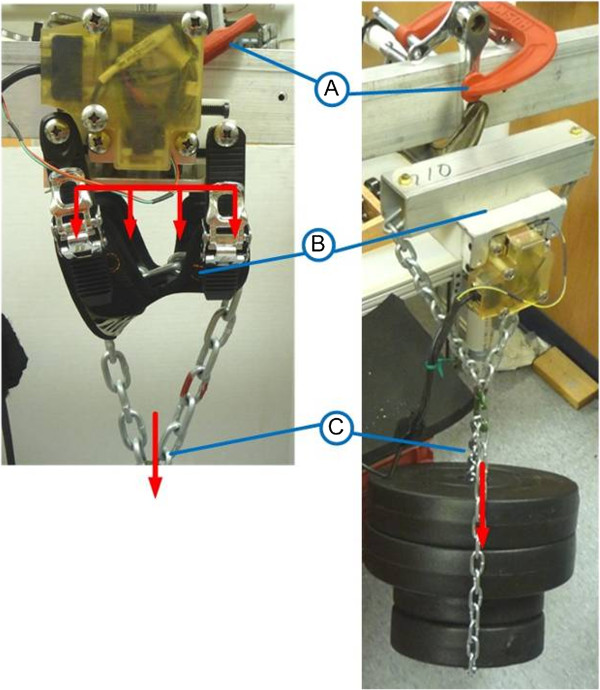
Static loading tension and compression for calibration of instrumented pedals: left and right respectively; A: mounting conditions identical to stationary bike; B: loading surface (s); C: static loading in line with load cell and foot placement.

**Figure 13:**
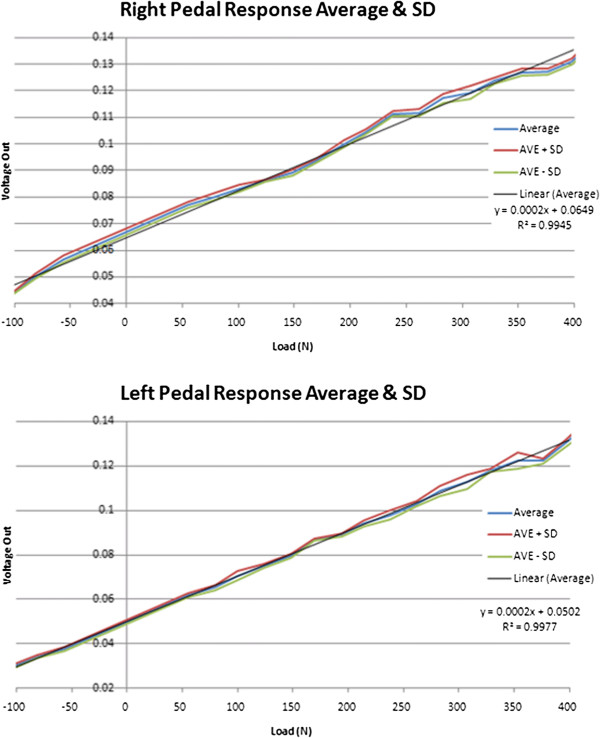
Voltage - load calibration curves obtained from static loading of instrumented pedals during compression and tension tests.

The Pedal assembly was calibrated and then validated using static loading conditions for compressive and tensile force measurement (420 N, 120 N) as shown in Figure 12. For compression testing the pedal was mounted via a pin joint to a crank arm and weights hung underneath, with a rigid bar contacting the footplate surface. This setup was important to also assess the impact of the moment bending on the crank raceway. The load was incrementally increased taking into account the weight of the rigid bar and chains. For tension the pedal was reversed and the load was applied to the ventral surface of the bindings just as in application of the ‘lifting’ force during the upstroke. Weights were allowed 30 seconds to settle once loaded, and between loads the resting voltage was also measured to check for drift. Results from these calibration tests, shown in Figure 13, are averaged for 4 loading trials per pedal.

#### ***IMU Calibration and validation using a specially design testbed***

**Figure 14:**
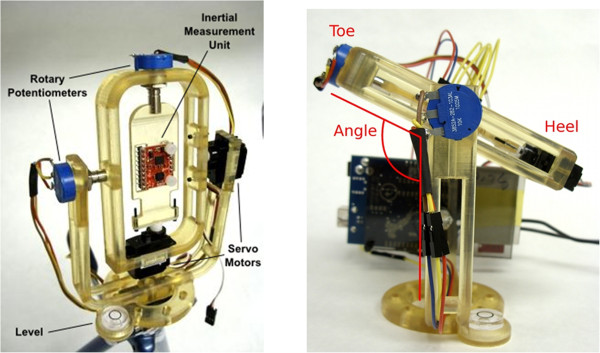
Two degree of freedom (DOF) testbed for IMU calibration and validation (left) and IMU testbed in a configuration emulating the pedal conditions (right).

A testbed was built for calibrating and validating the pedal IMU as shown in Figure 14. The test-bed is a two-degree of freedom structure similar to a universal joint. The IMU is placed on a platform in the middle of the structure while each one of the revolute joints is equipped with a servo motor for applying controllable angular motions at this joint and a rotary potentiometer for measuring the joint’s angular position and velocity. An Arduino Duemilanove microcontroller (http://www.arduino.cc) was added to the assembly to control the servomotors and to collect data from the IMU and rotary potentiometers.

**Figure 15:**
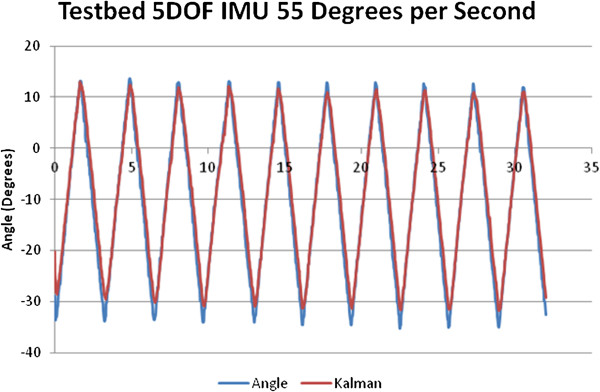
Pedal angle calculated using the IMU measurement and the Kalman filter (line in red) vs. the pedal angle measured using the potentiometer of the IMU testbed.

In calculating the angle of the pedal the signals needed from the 5 DOF IMU were those from the X and Z accelerometers and the Y gyroscope. A Kalman filter algorithm used the accelerometer and gyroscope readings to provide an estimate of the pedal angle based on the method presented in [40]. Figure 15 shows an example of the IMU validation tests. The IMU readings/Kalman filter algorithm produced pedal angles that matched very accurately those measured independently by the potentiometer on the IMU test bed.

### Rider testing

Characterization of the VRACK instrumentation beyond bench testing is necessary to validate both the hardware and the software with individuals riding the bicycle. In this paper we present data obtained from the instrumented pedals when they were manually moved and with individuals riding the bicycle. In a recent study we have also validated VE features such as optic flow effects on riders cycling performance [42].

The focus of the rider experiments presented in this section was to validate the data (i.e. pedal angle and pedal forces) obtained using the left and right instrumented pedals during a cycling session. During these experiments we concurrently collected kinematic data with both the VRACK IMU and a Peak Motus motion capture system and compared them. Kinetic data were collected using the pedal force sensors and compared with similar data reported in literature.A six camera Peak Motus motion capture system was used to record the kinematics at 60 Hz during pedaling on a recumbent bicycle (Biodex, SRC). Simultaneously the VRACK system collected the data from the bicycle’s pedal IMU at 100 Hz using a real-time Labview program as described in Figure 4. Data from the Peak were re-sampled to 100Hz to match the IMU sampling frequency. Data were synchronized by matching the peak pedal angle with the first five seconds of the trial. The pedal marker data from the Peak Motus system were used to measure the pedal orientation and cycling RPM. The marker data were processed and gaps were filled using smoothing spline function post collection. The orientation of the line joining the marker in the front and back of the pedal was calculated to obtain the pedal angle. The data from the VRACK system were analyzed to extract the pedal orientation and forces.

#### ***Experiment 1 - hand driven pedal motions***

**Figure 16:**
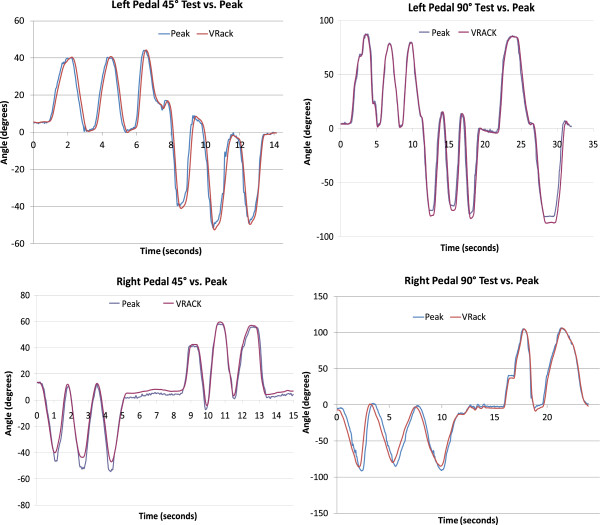
Experiment 1 - Hand driven pedal motions - Pedal angle calculated using VRACK’s IMU measurement vs. pedal angle measured using the Peak Motus motion capture system.

The bike pedals were instrumented with three markers each on the front, middle and back. The middle marker had an extension so it would not be obscured by the front and back markers. The experimenter knelt on the left side of the bike and slid his hand into the right pedal when collecting data for the right pedal. The crank was placed at top dead center. Data were collected in two separate trials for each pedal. The pedal was turned manually, clockwise three times, and then counter clockwise three times, from 0–90 degrees in one trial and then from 0–45 degrees in a second trial. The same procedure was repeated for the other pedal. There was good agreement between the pedal angles measured by the Peak and the IMU on the VRACK as shown in Figure 16.

#### ***Experiment 2 - riders biking in the VE***

Five healthy participants (18–35 years) without any mobility, functional, or cardiovascular disorders provided their informed consent and participated in the study. The study was approved by the Institutional Review Board at the UMDNJ.

Following an orientation to the protocol, subjects were seated on the bike and positioned with 50 degrees of knee flexion when the pedal was at bottom dead center and parallel to the ground. The power on the bike was set to a constant 20 watts. Subjects warmed up by pedaling at a comfortable speed for 3 minutes. They were instructed to pedal at their slow and comfortable speeds, keeping both hands on the handle-bars, and looking in front of them. Pedals were instrumented with three reflective pedal markers; one each on the center, back, and front lateral edge of each pedal. *Data were collected for three trials of thirty to forty-five seconds each*.

**Figure 17:**
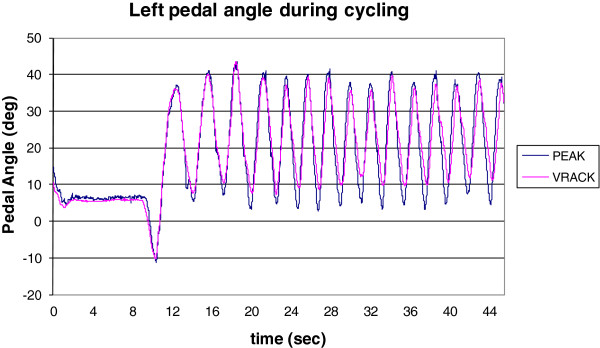
Experiment 2 - Rider biking in the VE at 30 RPM - Pedal angle calculated using VRACK’s IMU measurement vs. the pedal angle measured using the Peak Motus motion capture system.

**Figure 18:**
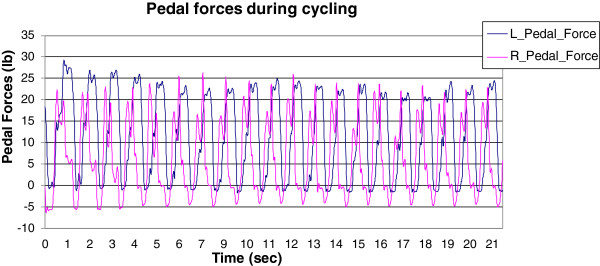
Experiment 2 - Rider biking in the VE at 55 RPM - Left and right pedal forces measured by the VRACK system.

A representative result is shown in Figure 17 where left pedal angles during a rider’s slow pedaling (30 RPM) are measured using the VRACKs’ IMU and the PEAK system. Both measurements demonstrate good agreement as shown below. Representative results from the forces collected by the VRACK are presented in Figure 18. The forces generated while the subject pedaled at 55 RPM indicate good correspondence between the two sides. The pattern of pedal forces is comparable but the magnitude of the pedal forces is lower than those reported in the literature [43,44]. The forces collected from VRACK showed a peak force of approximately 115 N (25 pounds as shown in the figure) when pedalling at 30 RPM with a workload of 20 Joules. In [43], where the authors used a more upright cycle and higher workload, reported, a peak pedal force of approximately 190 N when pedaling at 25 RPM with a workload of 80 J. Although in [44] the authors used a recumbent bike their participants were adolescents who cycled at a higher RPM (60) and they reported peak pedal forces of approximately 200 N. The magnitude of pedal forces generated in this setup were slightly smaller compared to [43] due to seating on the recumbent cycle and lower power setting used for the study. Therefore the lower peak forces collected on the VRACK can be explained by lower workloads, cycling speed and rider-to-bike biomechanics.

## Conclusions

In this paper the virtual reality augmented cycling kit (VRACK), a mechatronic rehabilitation system with an interactive virtual environment, was presented. VRACK consists of sensorized pedals, handlebars and a heart rate monitor interfaced with a virtual biking environment. VRACK was designed to benefit users with riding asymmetry by using quantitative measures to dynamically direct their attention. Work with individuals post-stroke who are present with fitness deficits and riding asymmetry is underway and the preliminary findings are encouraging [45].

VRACK and its modules offer several possibilities to augment existing home-based exercise equipment or used separately as stand-alone modules depending on what exercise is prescribed. The hydraulic chamber design of the handles could also be separated into smaller arrays of sensing regions to monitor more surfaces across the hands for either healthy or impaired individuals. For low ranges of upper extremity loading it could function as a computer interface/virtual reality device or dexterity training tool. For medium ranges of loading the design can be modified to map force distribution for power grasping pull tasks like lifting a briefcase or moving objects.

The VRACK system includes signals from 15 different sensors. This large number of sensors necessitates the need for robust signal acquisition hardware and software with proper filtering. Making the handle and pedal modules wireless will ease installation and reduce potential tripping hazard from the tethered modules. This will also be pragmatic for groups of VRACK systems to operate side by side for group exercise sessions in a clinical setting. Ultimately the VRACK’s relevance will be established when riders can modify their cycling kinetics from asymmetrical to symmetrical patterns, improve their fitness and more importantly transfer the benefits from training in the VE to real world mobility.

## Competing interests

The authors declare that they have no competing interests. JL and JED are co-owners of VRehab, the company that received the STTR.

## Authors’ contributions

JED, JL: conceived the project, participated in the VRACK design and validation. RGR, MLS, CM: carried out the design, fabrication, bench testing of the VRACK system. VKG, JED: carried out the human subject tests and performed data analysis. All authors read and approved the final manuscript.
